# Biological contamination of macrobenthic communities in seminatural streams: implications for monitoring habitat quality in *Isoëtes malinverniana* stands

**DOI:** 10.1007/s10661-026-15738-8

**Published:** 2026-07-31

**Authors:** Daniele Paganelli, Martina Tarascio, Thomas Abeli

**Affiliations:** University of Pavia, Pavia, Italy

**Keywords:** Alien species, Abundance contamination index, Biocontamination index, Habitat quality, Irrigation canals, Secondary hydrographic system

## Abstract

Seminatural streams and irrigation canals can act as important refuges for freshwater biodiversity in intensively managed landscapes, but they are also highly vulnerable to habitat degradation and biological invasions. In the northwestern Po Valley (Northern Italy), this secondary hydrographic network hosts the last remaining populations of *Isoëtes malinverniana*, a critically endangered endemic quillwort that has undergone a severe decline in recent decades. Despite the ecological relevance of benthic macroinvertebrates as indicators of habitat conditions, their communities have never been investigated in relation to the distribution of this species. We characterized macrobenthic communities in nine seminatural streams, including current and historical sites of *I. malinverniana* occurrence and one recent reintroduction site. Macroinvertebrates were sampled in spring 2025 together with basic physicochemical variables. Community composition, diversity patterns, and the occurrence of alien taxa were compared among streams to explore their relationship with the presence of *I. malinverniana*. Benthic assemblages were dominated by insects, particularly Chironomidae, while mollusks and crustaceans contributed less to total abundance. Only four alien taxa were recorded, yet their abundance varied markedly among streams. Streams hosting extant populations of *I. malinverniana* showed low levels of alien macroinvertebrates and environmental conditions indicative of limited disturbance, whereas historical sites of species loss and the reintroduction site exhibited higher proportions of alien taxa and signs of habitat alteration. Although overall macroinvertebrate diversity was higher where *I. malinverniana* is absent, this pattern was largely driven by alien species. These results indicate that macrobenthic community composition, particularly the presence of alien taxa, reflects environmental gradients critical for the persistence of *I. malinverniana*. Integrating macrobenthic assessments into monitoring programs may therefore support conservation and management actions for this critically endangered species.

**Supplementary Information:**

The online version contains supplementary material available at 10.1007/s10661-026-15738-8.

## Introduction

Traditionally, freshwater research has primarily concentrated on rivers, streams, and lakes, while relatively little attention has been paid to smaller natural or artificial habitats, such as agricultural canals and secondary hydrographic networks (Williams et al., 2003). Consequently, information regarding these anthropogenic freshwater environments remains limited (Valisena & Doretto, 2026). However, in landscapes where natural habitats are scarce, such man-made water bodies play a crucial ecological role by serving as substitute ecosystems, providing shelter for local biodiversity (Chester & Robson, 2013; Chiorino et al., 2024; Montanari et al., 2020; Rappocciolo et al., 2026). This is particularly evident in the huge irrigation system composed of > 4000 km of irrigation channels of the western Po Valley (Northern Italy). These environments are mainly anthropogenic water courses with oligotrophic to mesotrophic slow-flowing water, artificially regulated and used for irrigation of the paddy fields in the area. Nevertheless, they support important wetland habitats protected by the European Union Directive 92/43/EEC “Habitats,” such as habitat 3260 “Water courses of plain to montane levels with the *Ranunculion fluitantis* and Callitricho-Batrachion vegetation” and habitat 6430 “Hydrophilous tall herb fringe communities of plains and of the montane to alpine levels” (Orsenigo & Corli, 2022). Here, endemic plant and animal species or taxa of conservation interest that had disappeared in other areas still survive. Examples are the common water moss *Fontinalis antipyretica* Hedw., the royal fern *Osmunda regalis* L., *Callitriche* sp. pl., the Po Valley lamprey *Lampetra zanandreai* Vladykov, and the Padanian goby *Padogobius bonelli* Bonaparte (Bogliani et al., 2017; Bracco et al., 2001). Moreover, such irrigation system represents the only stepping-stone habitats for the critically endangered endemic quillwort *Isoëtes malinverniana* Cesati & De Not.

Intensive anthropogenic activities produce substantial ecological pressures and disturbances on this wetland system (Bo et al., 2025). Increased nutrient loads in water are due to runoff of fertilizers from intensive farming and sometimes to the release of urban wastewaters. This has progressively changed originally oligotrophic water into mesotrophic habitats (Viaroli et al., 2013). An excessive nutrient load has overall negative impacts on either water or substrate quality, through the accumulation of nitrates and phosphates, with long-standing effects (Severini et al., 2023). Moreover, the growth of macrophytes promoted by water nutrient enrichment requires more frequent canal management like dredging and mechanical cleaning that strongly affect biodiversity (Willby et al., 2001).

Among the major stressors affecting freshwater ecosystems, invasive alien species (IAS) represent a significant threat, and secondary hydrographic systems can often facilitate their dispersal (Nilsson et al., 2010; Rahel, 2002).

Despite the so-called “paradox of biological invasions” described by Fenoglio et al. (2016), which highlights the relative scarcity of invasive insects in freshwater systems, other aquatic invertebrates (i.e., crustaceans and mollusks) constitute some of the most invasive taxa. Their success is largely attributed to biological traits characteristic of efficient colonizers (Bij de Vaate et al., 2002) and their capacity to inhabit both lentic and lotic environments (Paganelli et al., 2015,2018; Pandolfi et al., 2017). When invasive species achieve high population densities, they have high ecological impacts, acting as biological pollutants or biocontaminants. These processes often result in biotic homogenization and consequent losses of native biodiversity (Arbačiauskas et al., 2008; Elliott, 2003).

Altogether, these pressures have been altering the habitat for many species, including *I. malinverniana*, a species that underwent a strong range decline in the last few decades (Christenhusz et al., 2017). Despite the recognized importance of benthic macroinvertebrates as ecological bioindicators for evaluating the status and potential changes of freshwater habitats, no studies have yet examined the macrobenthic communities inhabiting channels colonized by *I. malinverniana*. Investigating these communities within the distribution range of the species may therefore provide valuable insights into the ecological suitability of these habitats for the species and help identify environmental changes that could adversely affect its persistence.

This study aims to investigate for the first time the macrobenthic composition in current and historical sites of *I. malinverniana* and in the only reintroduction site with the following objectives: (1) characterize the composition of macroinvertebrate communities, (2) explore possible ecological relationships between the presence of *I. malinverniana* and the biological pollution of the channels, and (3) create a baseline for the future use of macrobenthos as an ecological monitoring tool in *I. malinverniana* conservation and management programs.

## Methods

### Study area and species

*Isoëtes malinverniana* is a fully aquatic quillwort endemic to the northwestern Po Valley, in Northern Italy, where about ten populations with a declining trend are present in Piedmont (provinces of Novara and Vercelli) and Lombardy (province of Pavia). The species is assessed as critically endangered (CR) globally and in the Red List of the Italian Flora (Christenhusz et al., 2017; Rossi et al., 2013) and protected at the European (Directive 92/43/EEC “Habitats”; Annexes II and IV) and local level (Piedmont and Lombardy). Nowadays, *I. malinverniana* occupies exclusively seminatural streams and artificial canals from the secondary hydrographic system comprised between the Ticino and the Sesia River valleys (Barni et al., 2013). The upstream part of the irrigation system (provinces of Vercelli and Novara, eastern Piedmont) is characterized by clear, oligotrophic running waters, characterized by slightly basic pH (7.5–7.8) and low nutrient content (81–105 µS/cm), flowing mainly from natural springs (Barni et al., 2013). The downstream part of the irrigation system (province of Pavia, Lombardy) receives water inputs from larger streams and rivers (including the Sesia, the Ticino and the Po rivers) and from the nearby paddies especially during the rice cultivation season (May–October); here water becomes progressively mesotrophic (electrical conductivity, EC 105–120 µS/cm) and occasionally turbid (Barni et al., 2013).

Consequently, *I. malinverniana* is more abundant in the upper Po Plain in eastern Piedmont and progressively less abundant toward the south in Lombardy, where the species has been declining for the last 20 years to a single wild population. Barni et al. (2013) showed that *I. malinverniana* has disappeared from channels with turbid waters, with higher EC related to an increase in nitrates and higher soil phosphorus and potassium content. Besides changes in water quality, other threats for the species include hydrological alterations due to mechanical dredging of irrigation channels and prolonged winter dry spells (Abeli et al., 2020; Barni et al., 2013).

### Sampling sites and design

In total, nine sites were selected in the Province of Vercelli and Novara in Piedmont and Province of Pavia in Lombardy (northwestern Italy, Table 1).

**Table 1:** Code and presence of *Isoëtes malinverniana* and water physical–chemical parameters of the nine target seminatural streams

Stream code	Presence of *I. malinverniana* (yes, no)	T (°C)	O_2_ (mg/L)	% saturation	pH	EC (µS/cm)
SP594	Y	12.30 ± 0.361	8.74 ± 0.010	83.57 ± 0.306	6.91 ± 0.047	143.17 ± 0.416
LEN	Y	12.90 ± 0.608	9.53 ± 0.096	92.60 ± 0.436	6.73 ± 0.026	180.73 ± 0.231
CMC	Y	14.53 ± 0.252	9.14 ± 0.040	91.87 ± 0.153	6.38 ± 0.044	132.40 ± 0.265
GSP	Y	15.13 ± 0.153	9.87 ± 0.036	100.33 ± 0.115	6.62 ± 0.078	88.47 ± 0.252
M	Y* reintroduced	15.80 ± 0.200	9.37 ± 0.110	94.70 ± 0.700	7.07 ± 0.017	258.67 ± 2.309
A	N	15.46 ± 0.057	10.79 ± 0.005	110.2 ± 0.173	6.83 ± 0.01	100 ± 0.01
B	N	17.60 ± 0.458	8.67 ± 0.491	95.53 ± 0.462	7.45 ± 0.144	250.0 ± 1.0
C	N	18.47 ± 0.058	8.17 ± 0.035	87.5 ± 0.300	7.26 ± 0.012	278 ± 0.000
D	N	18.30 ± 0.265	9.88 ± 0.085	105.43 ± 0.379	7.42 ± 0.113	202.63 ± 0.306

**Fig. 1:**
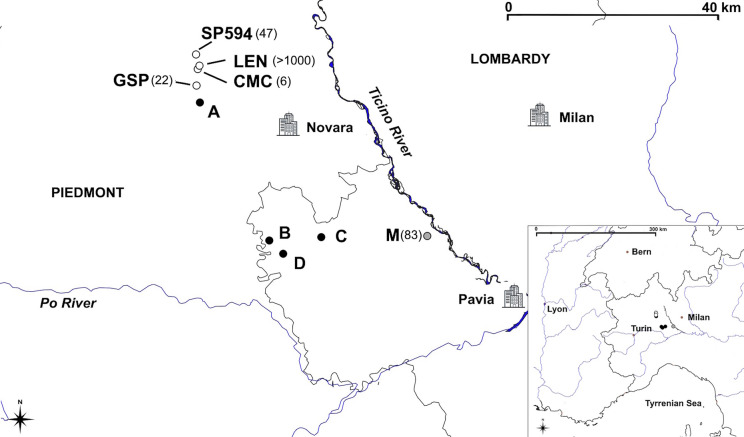
Study area and location of nine sampled sites. White spots indicate extant populations of *Isoëtes malinverniana*; black spots indicate historical sites of occurrence of the target species; the gray spot (M) indicates a reintroduction site. Numbers in parentheses indicate *I. malinverniana* population size

In particular, the streams were selected according to the presence of *I. malinverniana*: four of them represented historical growing sites that did not host the species anymore; in the other four, the plant was naturally present, and in one of them, the plant was recently experimentally reintroduced in 2022 (Fig. 1, Table 1). The four sites of occurrence of the species represent almost half of the ten known populations of this rare quillwort.

All nine seminatural streams are characterized by similar ecological conditions, with sandy-muddy to gravel bottom and moderate running water.

In each seminatural stream, between the end of April and beginning of May 2025, the sampling protocol was conducted collecting three separate subsamples of macrozoobenthos along 10 m transect consisting of homogeneous habitat, mainly formed of a mix of small gravel, sand, and mud which reflects the entire characteristics of all the seminatural streams under study, using a modified net (950 μm mesh size) equipped with a square frame (22 × 23 cm, for an area of 0.0506 m^2^).

In each seminatural stream, we also recorded water environmental variables, namely, pH, conductivity, temperature, and oxygen concentration estimating the percentage of oxygen saturation in the water using a multi probe HACH HQ40d.

Biological samples were then preserved in 70% ethanol and transferred to the laboratory for identification at the species level (when it was possible) and counting.

Once the macrobenthic community was identified, we calculated the level of biodiversity using the Shannon–Wiener diversity index (*H*′) in two versions: in the first, we included the alien species, while in the second version, we used only the local community in order to highlight the role of alien species in the community; finally, the level of biocontamination in each stream was calculated using the site-specific biocontamination index (SBCI, Arbačiauskas et al., 2008). The SBCI is derived from two subindices, namely, the abundance contamination index (ACI) and the richness contamination index (RCI). The ACI is calculated as the ratio between the number of specimens of alien taxa and the total number of specimens in a sample; considering the level of identification we obtained, we decided to calculate the RCI subindex as the ratio between the total number of alien families and the total number of families as proposed by MacNeil et al. (2010).

SBCI scores are divided into five classes, and their scores vary from 0 (no biocontamination, high status) to 4 (severe contamination, bad status)—matching the common implementation strategy for the EU Water Framework Directive (2000/60/EC) (European Community, 2000).

Also, considering the aim of the study, the characteristics of the seminatural stream, the sampling protocol applied, and the level of identification obtained, we decided to define the ecological quality of the studied stream applying the Italian version of the extended biotic index (EBI, Woodiwiss, 1978), namely, the “Indice Biotico Esteso” (IBE), as modified by Ghetti (1986) for Italian water courses which was used up to 2006 for the definition of the biological quality before the introduction of the Water Frame Directive 2000/60 EC.

### Data analysis

The biological data was before transformed into a Bray–Curtis dissimilarity matrix, and then, the difference in composition of the macrobenthic community and the difference in alien species density in each seminatural stream were analyzed using permutational multivariate analysis of variance (PERMANOVA) based on a one-factor design with “stream” as the random factor and then followed by the Monte Carlo pairwise tests. Compared to traditional parametric analysis of variance, the PERMANOVA has the advantage that the assumptions of normality and homoscedasticity are not mandatory, so it appears to be the most suitable analysis with ecological datasets. Furthermore, to interpret the PERMANOVA results, the homogeneity of multivariate dispersion among streams was assessed using PERMDISP analysis.

After aggregating the three subsamples for each stream, differences between streams were then represented using an nMDS graph (stress value<0.2) and, using a SIMPER analysis, we defined the contribution of the species in the composition of the community of each seminatural streams.

Moreover, the differences between the biotic indexes (i.e., Shannon–Wiener diversity index (*H*′), the abundance contamination index (ACI) and the richness contamination index (RCI)) of each community were tested by means of an ANOVA test followed by Tukey tests.

Finally, to highlight a relation among the recorded environmental variables, IBE, ACI, and RCI scores and the abundance of *I. malinverniana* in the studied streams, a PCA was conducted on the environmental and biological variables with five principal components (PCs) extracted.

All the statistics were performed using PRIMER v6 and PERMANOVA + and Minitab 18.

## Results

**Fig. 2:**
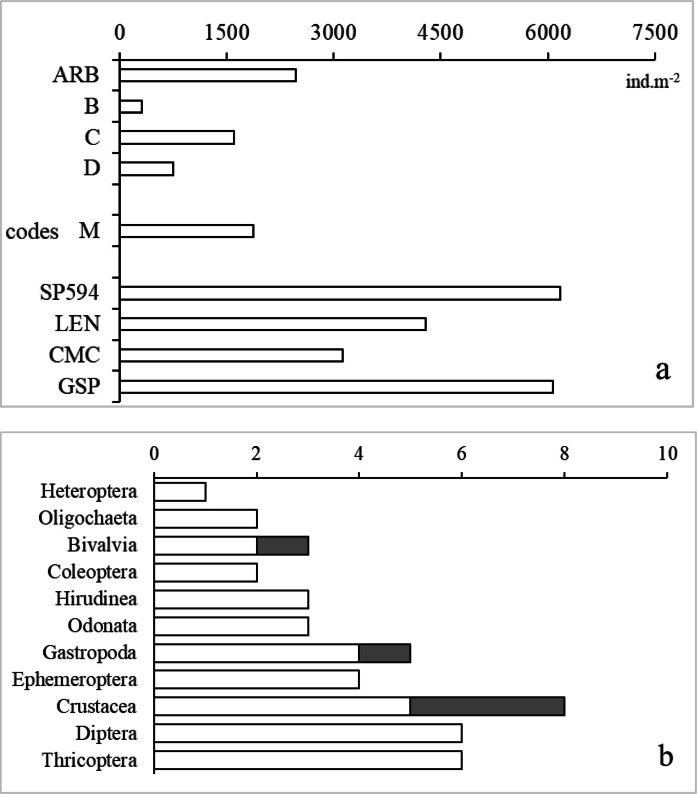
Macroinvertebrate community collected in the nine studied seminatural streams. **a** Total abundance of macroinvertebrates (ind. m^2^). **b** Composition of the community collected: Dark gray bars indicate the alien species while white bars indicate the number of native *taxa* (see Fig. 5 for the list of alien species detected in the study area)

Overall, in the nine seminatural streams, the highest density of macrobenthic taxa was found in SP594 while the lowest was recorded in D (Fig. 2a). Regarding the composition of the community, it was mainly constituted of insects (58%), followed by mollusks (15.8%) and crustaceans (13%) (Fig. 2b).

**Fig. 3:**
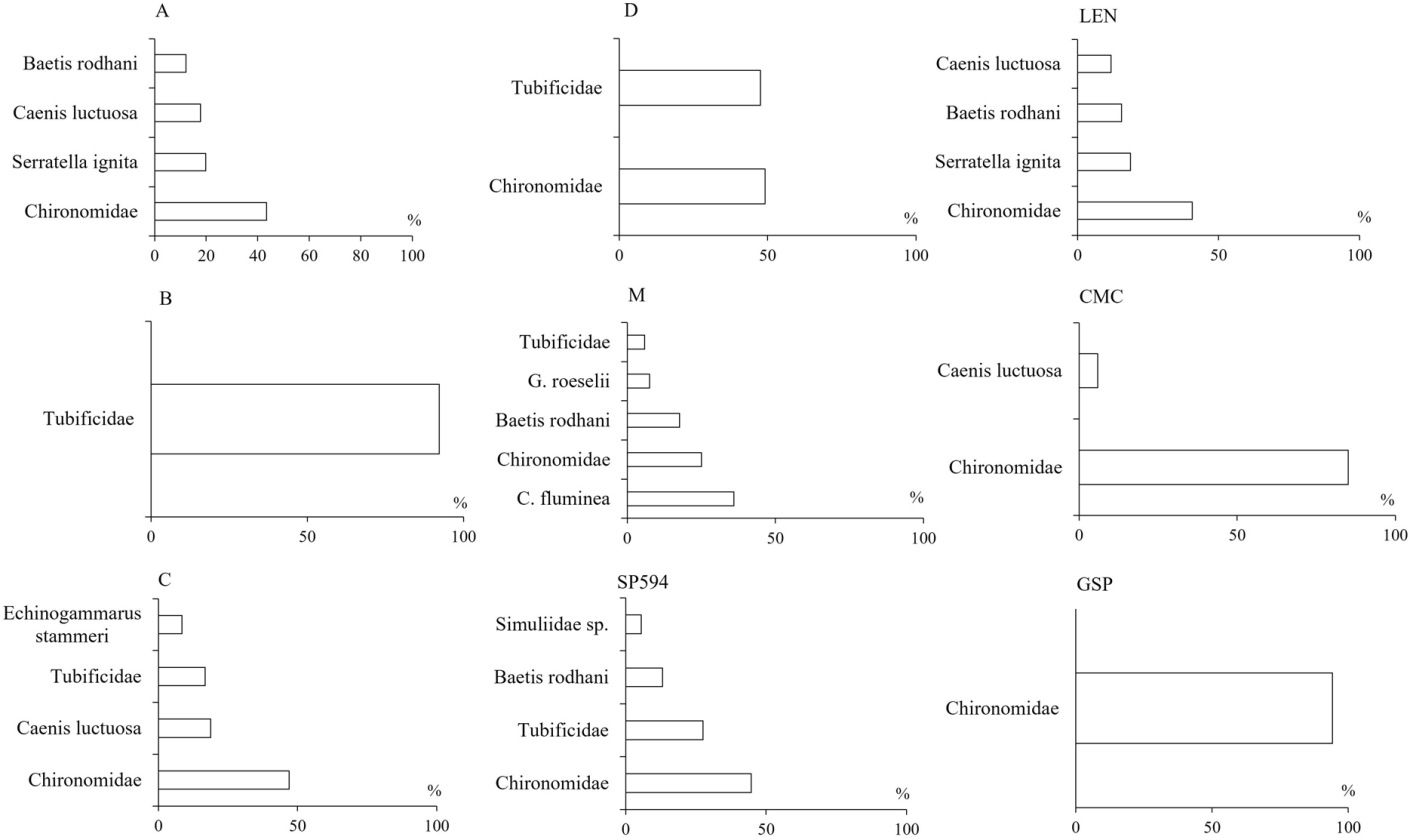
SIMPER results (% contribution of the main *taxa*) of the macrobenthic community analyzed in the nine seminatural streams. For the codes of the streams, see Table 1

Analyzing the community of each seminatural stream by means of the SIMPER analysis, it emerged that almost all of them are dominated by specimens of the Chironomidae family, apart from M where the most abundant is the invasive alien mollusk *Corbicula fluminea* (Fig. 3).

**Fig. 4:**
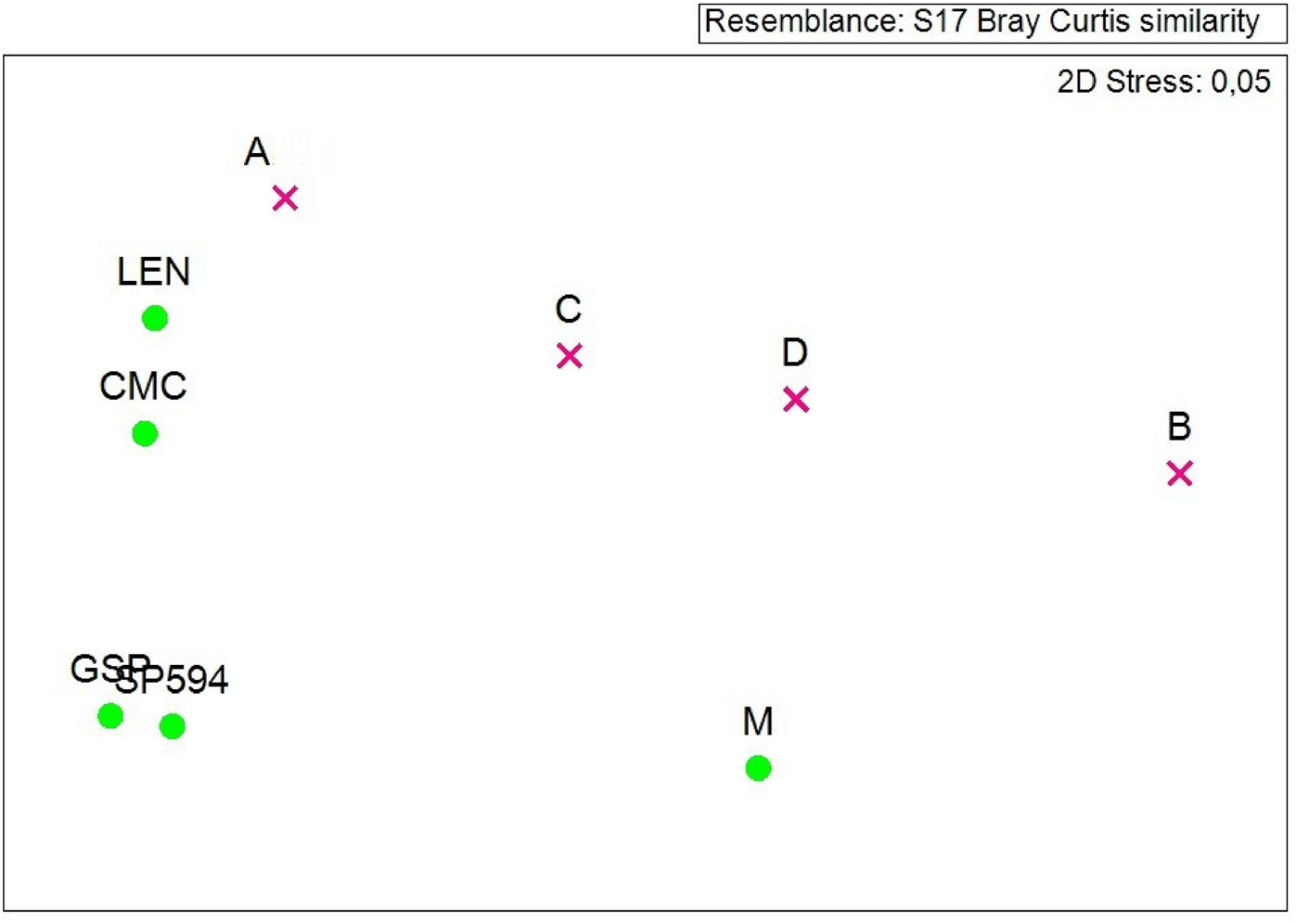
nMDS of the community. Current sites of occurrence of *Isoëtes malinverniana* (green circle: LEN, CMC, GSP, SP594, and a reintroduction site M); historical sites of occurrence (red cross: A, B, C, D) are represented. For the codes of the streams, see Table 1

Despite the high abundance of specimens belonging to this family in all the seminatural streams, a deeper analysis using the PERMANOVA routine followed by the pairwise Monte Carlo tests highlighted a significant difference between streams (Pseudo-*F* = 2.894; df = 8; *p* < 0.001; Supplementary Material 1; Fig. 4). However, PERMDISP revealed significant heterogeneity of multivariate dispersion among streams (*F* = 10.774, *p* = 0.013).

Dividing the nine seminatural streams into two groups related to the presence of *I. malinverniana* and analyzing the macrobenthic community with the PERMANOVA routine, a statistical difference emerged between them (Pseudo-*F* = 3.55; df = 1; *p* = 0.002) supported by PERMDISP (*F* = 0.1854, *p* = 0.716).

**Fig. 5:**
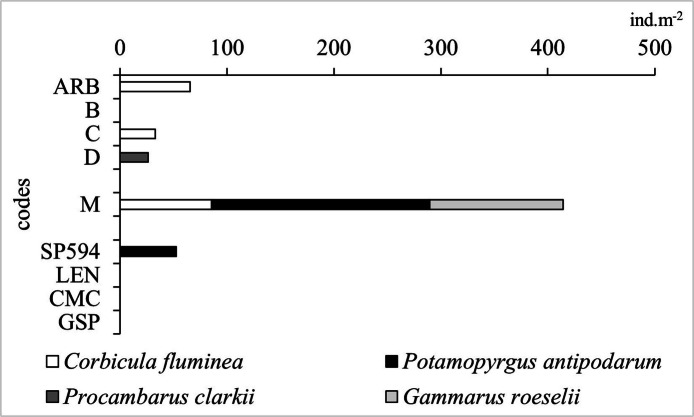
Abundance of alien species in the nine studied seminatural streams. Current sites of occurrence of *Isoëtes malinverniana* (LEN, CMC, GSP, SP594), historical sites of occurrence (A, B, C, D), and a reintroduction site (M) are represented. For the codes of the streams, see Table 1

The next step was to analyze the macrobenthic community only considering the presence of alien species and the PERMANOVA routine still indicated a significant difference between streams (Pseudo-*F* = 5.1348; df = 4; *p* < 0.001; Fig. 5), confirmed by PERMDISP test (*F* = 4.0378, *p* = 0.644). The Monte Carlo tests indicated that this result was mainly due to differences in SP594 vs C, SP594 vs A, and D vs C (Supplementary Material 2).

Out of the 38 species or families identified, only four of them were alien, namely, the mollusks *Corbicula fluminea* (Müller, 1774) and *Potamopyrgus antipodarum* (Gray, 1843) and the crustaceans *Procambarus clarkii* (Girard 1882) and *Gammarus roeselii* Gervais, 1835 (Fig. 5).

The presence of the four alien species generated significant differences both in ACI (*F* = 4.19; df = 8; *p* = 0.006) and RCI (*F* = 8.05; df = 8; *p* < 0.001) scores.

**Fig. 6:**
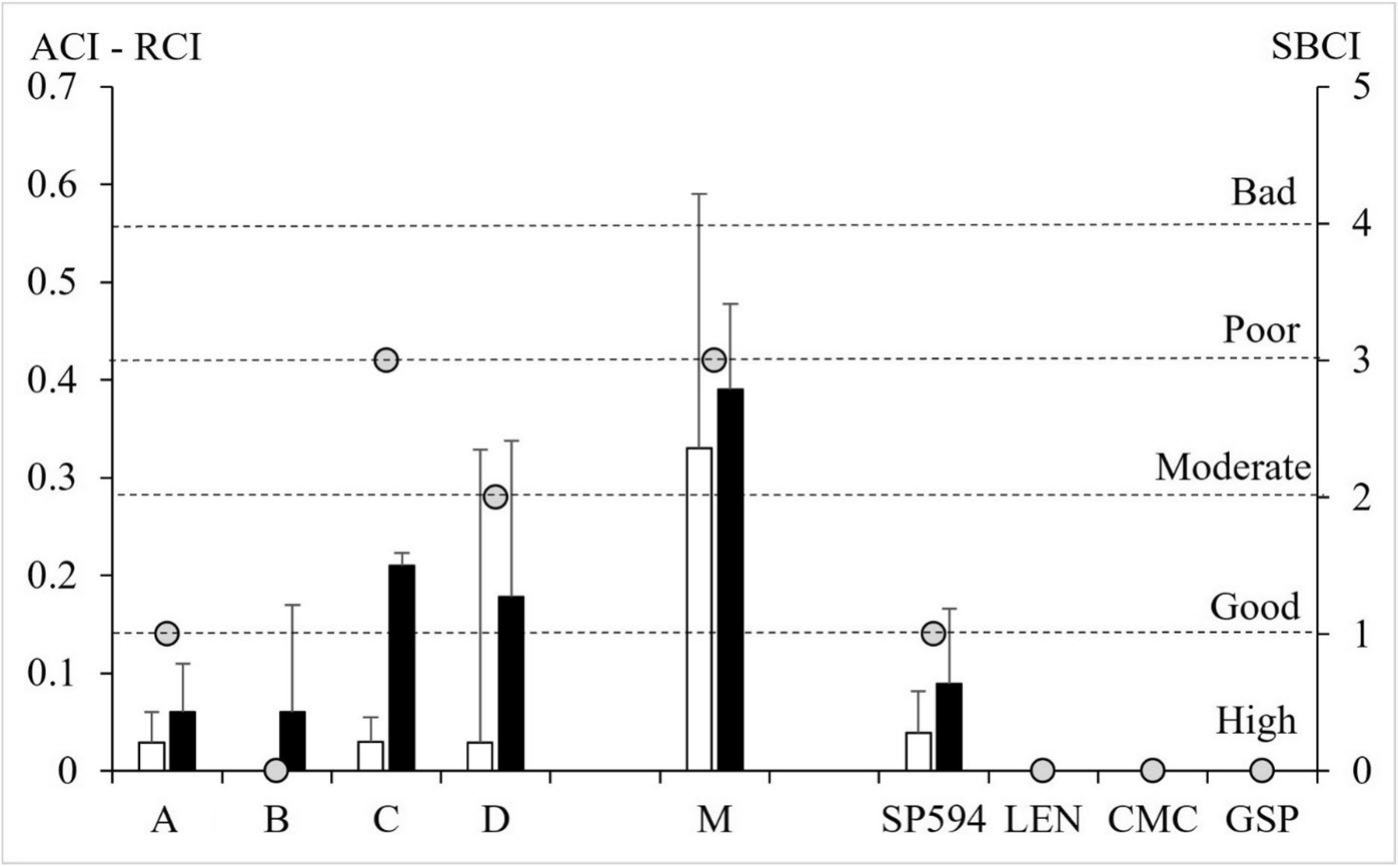
Mean scores (± standard deviation) of ACI (white bars), RCI (black bars), and SBCI (gray dots) indices. SBCI classes are 0 (no contamination, high status), 1 (low contamination, good status), 2 (moderate contamination, moderate status), 3 (high contamination, poor status), and 4 (severe contamination, bad status). Modified from Arbačiauskas et al. (2008). Current sites of occurrence of *Isoëtes malinverniana* (LEN, CMC, GSP, SP594), historical sites of occurrence (A, B, C, D), and a reintroduction site (M) are represented. For the codes of the streams, see Table 1

Communities with the lowest level of contamination (SBCI = 0) were LEN, GSP, CMC, and B, while the communities with the highest value (SBCI = 3) were C, only due to the relative abundance of *C. fluminea*, and M where there were three out of four alien species present, namely, *C. fluminea*, *P. antipodarum*, and *G. roeselii* (Fig. 6).

Comparing the level of contamination with the ecological quality of the nine seminatural streams does not indicate a strong correspondence between these indices (Table 2).

**Table 2:** Comparison between SBCI results and ecological quality defined using the extended biotic index of the nine seminatural streams. Current sites of occurrence of *Isoëtes malinverniana* (LEN, CMC, GSP, SP594), historical sites of occurrence (A, B, C, D), and a reintroduction site (M) are represented. For the codes of the streams, see Table 1

Seminatural stream	SBCI results	Level of contamination	IBE	Ecological quality
SP594	1	Low contamination	5–6	IV-III
LEN	0	No contamination	9	II
CMC	0	No contamination	6–7	III
GSP	0	No contamination	6	III
M	3	High contamination	6–7	III
A	1	Low contamination	8	II
B	0	No contamination	5–4	IV
C	3	High contamination	7	III
D	2	Moderate contamination	5–4	IV

**Fig. 7:**
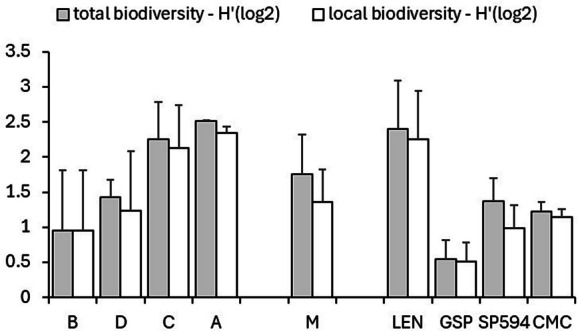
Total and local biodiversity (mean value ± standard deviation) of the community in the nine studied seminatural streams. Current sites of occurrence of *Isoëtes malinverniana* (LEN, CMC, GSP, SP594), historical sites of occurrence (A, B, C, D), and a reintroduction site (M) are represented. For the codes of the streams, see Table 1

The level of biodiversity (Shannon index) is higher in those streams where *I. malinverniana* is not present anymore and, also, it is interesting to note that, in all the nine seminatural streams, the level of biodiversity is always slightly higher taking into consideration alien species (Fig. 7).

Finally, a PCA was performed: the first component (PC1) accounts for the highest variation, explaining 45% of the total variance in the data. The cumulative variance after three axes reached 81.8%, suggesting that the first three components capture a substantial portion of the variability (Table 3).

**Table 3:** Eigenvectors of the PCA performed on eight variables

Variables	PC1	PC2	PC3	PC4	PC5
IBE	0.298	0.494	−0.107	−0–325	−0.580
ACI—abundance contamination index	−0.253	0.513	0.461	0.219	0.095
RCI—richness contamination index	−0.42	0.376	0.219	0.070	−0.047
Population size of *I. malinverniana*	0.253	0.467	−0.435	−0–272	0.433
Water temperature (°C)	−0.399	−0.244	−0.003	−0.500	−0.454
Dissolved oxygen (mg/L)	0.237	−0.017	0.593	−0.611	0.255
pH	−0.443	−0.095	−0.166	−0.372	0.438
Conductivity (µs/m)	−0.443	0.254	−0.361	−0.071	−0.045

**Fig. 8:**
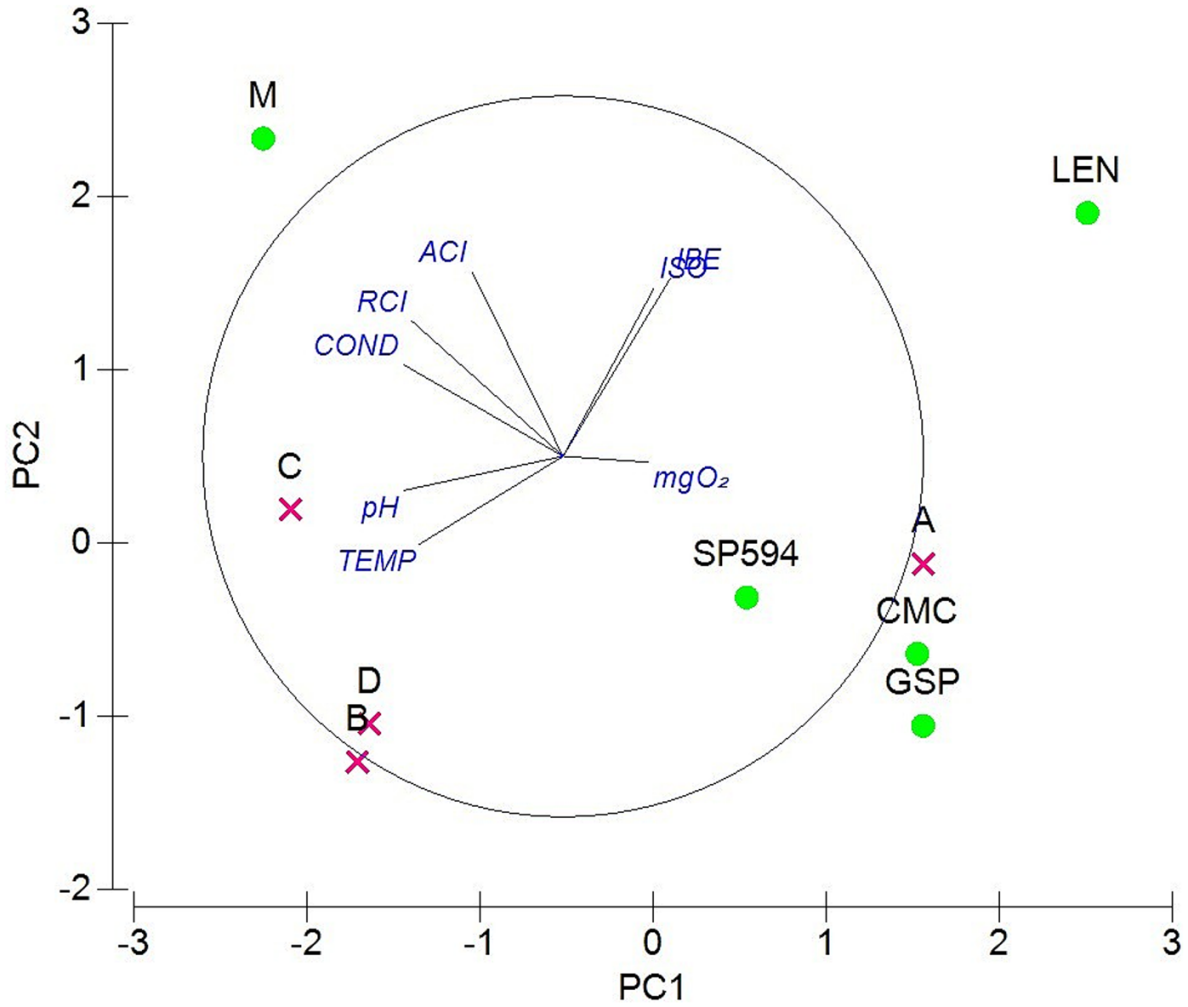
Spatial distribution along the first two axes of the studied seminatural streams and the variables used in the PCA. Current sites of occurrence of *Isoëtes malinverniana* (green circle: LEN, CMC, GSP, SP594, and a reintroduction site M) and historical sites of occurrence (red cross: A, B, C, D) are represented. For the codes of the streams, see Table 1

In the biplot, the spatial distribution along the two axes of the seminatural streams indicates that LEN, A, and CMC cluster are positively related on PC1 with variables like dissolved oxygen and weaker association with TEMP and pH. On the other hand, sites such as B, C, and D lie on the negative side of PC1, indicating inverse relationships with these variables and potentially lower dissolved oxygen or higher temperatures. M is more strongly separated along PC2, suggesting it may be influenced more heavily by variables like IBE, ISO, and ACI (Fig. 8).

## Discussion

This study analyzes for the first time the macrobenthic community in sites of occurrence of the critically endangered endemic quillwort *Isoëtes malinverniana*, compared to sites where the species has disappeared. On one side, the study of macrobenthos is globally recognized as a practical cost-effective bioindicator tool that can highlight long-term patters of habitat quality and integrity (Water Framework Directive—WFD 2000/60/EC) and can therefore be used for monitoring the trend in the status of *I. malinverniana* sites and habitat. On the other side, like other Isoëtid species, *I. malinverniana* is suspected to strongly influence the substrate where it occurs in dense stands, through the capacity of oxidizing the rhizosphere (Rørslett & Brettum, 1989), which in turn is expected to affect the macrobenthic community at such sites.

*Isoëtes malinverniana* has experienced a drastic decline in the last 30 years associated with changes in water quality and channel management of its seminatural habitat (Barni et al., 2013), especially in the southern part of its range, where the effects of intensive agriculture are more important than in the northern part (Fazzone & Marchesi, 2025). These changes are partially reflected in the different assemblage of macrobenthic taxa among sites of occurrence and historical sites of the target species. *Isoëtes* species thrive in oligotrophic waters in which plant and animal communities are typically poor (Wang et al., 2021). The analysis of simple physicochemical variables confirms the observation made by Abeli et al. (2012), i.e., *I. malinverniana* has disappeared from channels with increased solutes (increased electrical conductivity), temperatures, and pH (Fig. 8).

These physicochemical variables seem to affect the macrobenthic fauna in the analyzed canals, with SBCI increasing and overall ecological quality decreasing in sites where *I. malinverniana* is not present anymore (Table 2; Fig. 8).

Analyzing the whole composition of the macrobenthic communities, PERMANOVA highlighted significant differences between streams, but the PERMDISP test suggested that differences detected by PERMANOVA may be partially influenced by unequal within-group variability and this indicates heterogeneous dispersion among streams, implying that part of the observed differences may be due to differences in multivariate variance rather than solely to shifts in centroid location.

However, considering the occurrence and abundance only of macrobenthic alien species, our results indicate substantial variability among streams which could be closely intertwined with environmental conditions and with the current distribution of *I. malinverniana*. These results are both supported by PERMANOVA analyses and PERMDISP tests.

Although only four nonindigenous taxa were recorded—*Corbicula fluminea*, *Potamopyrgus antipodarum*, *Procambarus clarkii*, and *Gammarus roeselii*—their presence nonetheless generated significant differences in both community structure and biocontamination indices, revealing early but measurable signs of biological pollution even in these relatively small watercourses.

Although alien species remain numerically limited in most sites, their ecological roles should not be underestimated.* Corbicula fluminea*, the dominant alien species in site C, is known for its ecological impacts, such as habitat reduction for other native bivalves (Bódis et al., 2016; Paganelli et al., 2018) and the overexploitation of environmental resources (Vaughn & Hakenkamp, 2001), strong bioturbation capacity, and its ability to alter sediment oxygen dynamics (Sousa et al., 2014). Similarly, *P. antipodarum* can reach very high densities and monopolize periphyton resources, indirectly reducing food availability for native grazers and contributing to functional homogenization of benthic communities (Bersine et al., 2008; Hall et al., 2003).

The presence of *G. roeselii* in the reintroduction site is also ecologically relevant. Although native to Eastern Europe, this amphipod behaves as a successful colonizer across Western Europe and now considered an “exotic species, well established” in Central Europe (Josens et al., 2005); in Northern Italy, this species’ distribution was restricted to the Sile River basin in the northeastern part of the country (Ruffo & Stoch, 2005), but more recently, its presence was detected in the Ticino river basin, thus demonstrating that this species is colonizing new environments (Paganelli et al., 2015; Pandolfi et al., 2017). Its strong affinity for coarse substrates and high tolerance to variable water quality make it a competitor for microhabitats potentially occupied by native taxa (Paganelli et al., 2020). Moreover, the sporadic presence of *P. clarkii*—an ecosystem engineer with major effects on sediment resuspension, macrophyte uprooting, and nutrient release—raises concern, even if densities were currently low.

Interestingly, overall Shannon diversity (*H*′) was slightly higher when alien species were included. This superficially positive effect reflects a well-documented ecological artifact: Early invasion stages increase local diversity while causing regional homogenization (Elliott, 2003; Rahel, 2002). In our study, sites that do not host *I. malinverniana* anymore displayed both a higher macrobenthic richness and at the same time a greater presence of alien taxa, confirming that diversity increases do not equate to ecological quality. Similar conclusions were also obtained by Chiorino et al. (2024) when they explored the relationship between the macrobenthic community composition and water quality in agricultural canals in Northern Italy.

Although the number of alien species was relatively low, their abundance and distribution could indicate degraded channels from less impacted ones. The mismatch between SBCI and the extended biotic index (IBE) in several sites highlights that biocontamination conveys information that traditional biotic indices may not capture, particularly in seminatural irrigation channels where alien mollusks and crustaceans can thrive despite apparently “moderate” ecological quality according to IBE.

The pattern of alien species distribution closely reflected the gradient of environmental alteration described for the irrigation network, with the highest levels of biocontamination (SBCI = 3) observed in sites C and M. These two streams are also those where water quality parameters especially conductivity and temperature indicate a greater degree of potential hydrological alteration. These results are in accordance with previous findings which underline how *C. fluminea* and *P. antipodarum* successfully proliferate in disturbed, nutrient-enriched, and physically homogenized channels (Paganelli et al., 2018; Spyra et al., 2015). The presence of multiple alien taxa in site M (reintroduction site of *I. malinverniana*) further highlights how rapidly such species colonize canals acting as ecological corridors and their presence could even deeply modify the freshwater community and environment (Havel et al., 2015; Tölgyesi et al., 2022).

Streams where *I. malinverniana* is still present (LEN, CMC, GSP, SP594) were instead characterized by either an absence or very low abundance of alien macrobenthic species (SBCI = 0–1). This pattern reinforces the idea that alien taxa are reliable indicators of habitat degradation in these systems.

The PCA confirmed this association by placing sites with high alien density (C, M, D) and higher ACI and RCI on the negative side of PC1 and PC2, correlating with higher conductivity and temperature and lower dissolved oxygen—conditions unsuitable for *I. malinverniana*. Conversely, sites with healthy quillwort populations clustered along axes associated with higher oxygen availability and lower solute loads. Thus, alien species not only signal ecological deterioration but also act as proxies for the environmental pressures that threaten *I. malinverniana*.

The PCA further confirmed that ACI and RCI are negatively correlated with *I. malinverniana* population size, while they are positively associated with higher conductivity and temperature—factors known to be unfavorable for *I. malinverniana* (Abeli et al., 2012; Barni et al., 2013).

Taken together, the two biocontamination metrics prove sensitive to the environmental gradients that also determine the distribution of *I. malinverniana*. Their strong correspondence with the presence/absence of the species suggests that SBCI and its subindices can serve as early-warning indicators of habitat degradation.

Studies on the association between *Isoëtes* taxa and macrobenthos are generally scarce and most relative to lakes. Monospecific *Isoëtes* communities are often characterized by a low number and low biomass of macroinvertebrates (Waters & San Giovanni, 2002). In New Zealand, pure stands of *I. alpinus* were less rich in terms of macrobenthic invertebrates than any other macrophyte community (Talbot & Ward, 1987). Similar results were found in Argentina in stands of *I. savatieri* (Epele et al., 2012). The latter study also highlights seasonal patterns in the benthic assemblages, an aspect that we did not investigate. Given the hydrological regime of these agricultural canals, seasonal fluctuations in the macrobenthic community could be predicted (Rappocciolo et al., 2026). However, following the observations of Megan et al. (2007) and Hepp and Santos (2009) regarding impacted systems, the broad ecological valence of the sampled taxa suggests these variations will manifest as shifts in abundance and population density rather than fundamental alterations in taxonomic composition. This aspect is even more relevant in a biopolluted context such as the seminatural canals.

Overall, our results underscore the importance of integrating indices—such as SBCI—when evaluating habitat suitability for endangered, oligotrophy-dependent species such as *I. malinverniana*.

In particular, the definition of the level of biocontamination could effectively be a complement and also an enhancement of the traditional ecological quality assessments (Arbačiauskas et al., 2008). The strong association between low contamination (SBCI = 0–1) and the persistence of *I. malinverniana*, coupled with the elevated SBCI values observed in sites where the species has disappeared or struggles to establish, underscores the vulnerability of this endemic quillwort to subtle yet progressive ecological alterations.

Incorporating biocontamination indices into long-term monitoring and management programs therefore could be a useful tool to understand the level of habitat degradation, guide restoration actions, and ensure the effective conservation of this critically endangered plant species.

## Conclusion

This study highlights the importance of seminatural streams for their support of local biodiversity, but it also underlines their role as potential vectors for the spreading of invasive alien species. This is particularly true for degraded habitats which are more prone to biological pollution.

The presence and abundance of alien macrobenthic taxa therefore represent a practical early indicator of habitat degradation in these small freshwater environments. Incorporating alien species–based metrics into long-term monitoring programs will not only enhance our understanding of ecosystems but will also improve the effectiveness of conservation interventions, including future reintroductions.

The role of seminatural streams as refuges for macrobenthic biodiversity is also related to the surrounding environments: in an area where anthropogenic pressure is very high, such as Northern Italy, the management of these freshwater environments is fundamental.

For example, the sediment cleaning or the cutting of submerged vegetation as well as riparian vegetation should be kept to a minimum.

Another important management action could be related to the fluctuation of the water level: To sustain a stable community, water level variation should be limited and moreover the presence of the water should be guaranteed during all year.

Finally, but not less important in such environmental context, the physical–chemical conditions should be suitable for supporting a freshwater community.

## Supplementary Information

Below is the link to the electronic supplementary material.

ESM1 (DOCX 17.9 KB)

ESM2 (DOCX 15.3 KB)

## Data Availability

No datasets were generated or analysed during the current study.

## Declarations

All authors have read, understood, and complied as applicable with the statement on “ethical responsibilities of authors” as found in the instructions for authors.
