# Supplementary material for: Biological contamination of macrobenthic communities in seminatural streams: implications for monitoring habitat quality in Isoëtes malinverniana stands
Source: Environ Monit Assess. 2026 Jul 31;198(8):896. doi: 10.1007/s10661-026-15738-8 (PMC13427863; doi:10.1007/s10661-026-15738-8)
Supplement: Supplementary file 1 — (DOCX 17.9 KB) [file 10661_2026_15738_MOESM1_ESM.docx]

**Supplementary Material 1.** Pairwise Montecarlo tests results. Ns: not significant; *: p<0.05, significant; **: p<0.005, very significant; ***: p<0.001, extremely significant. For the codes of the streams, see table 1

| Groups | t | p(perm) | perms | p(MC) | Statistical significance |
| --- | --- | --- | --- | --- | --- |
| SP594, CMC | 1.1447 | 0.281 | 10 | 0.321 | ns |
| SP594, LEN | 1.3424 | 0.107 | 10 | 0.185 | ns |
| SP594, GSP | 1.1851 | 0.290 | 10 | 0.297 | ns |
| SP594, D | 1.8157 | 0.109 | 10 | 0.067 | ns |
| SP594, B | 1.3092 | 0.095 | 10 | 0.199 | ns |
| SP594, M | 1.0139 | 0.477 | 10 | 0.392 | ns |
| SP594, C | 1.3858 | 0.104 | 10 | 0.173 | ns |
| SP594, A | 1.4937 | 0.099 | 10 | 0.143 | ns |
| CMC, LEN | 1.5074 | 0.211 | 10 | 0.146 | ns |
| CMC, GSP | 1.5062 | 0.192 | 10 | 0.162 | ns |
| CMC, D | 2.3846 | 0.087 | 10 | 0.041 | * |
| CMC, B | 1.7396 | 0.094 | 10 | 0.076 | ns |
| CMC, M | 1.5462 | 0.102 | 10 | 0.098 | ns |
| CMC, C | 1.2768 | 0.329 | 10 | 0.206 | ns |
| CMC, A | 1.4942 | 0.084 | 10 | 0.151 | ns |
| LEN, GSP | 2.6890 | 0.110 | 10 | 0.024 | * |
| LEN, D | 4.0249 | 0.104 | 10 | 0.004 | ** |
| LEN, B | 2.0794 | 0.093 | 10 | 0.026 | * |
| LEN, M | 1.7156 | 0.112 | 10 | 0.088 | ns |
| LEN, C | 1.9672 | 0.105 | 10 | 0.045 | * |
| LEN, A | 1.5408 | 0.207 | 10 | 0.125 | ns |
| GSP, D | 4.8565 | 0.107 | 10 | 0.003 | ** |
| GSP, B | 2.1787 | 0.104 | 10 | 0.023 | * |
| GSP, M | 1.8819 | 0.088 | 10 | 0.049 | * |
| GSP, C | 2.6692 | 0.106 | 10 | 0.012 | ** |
| GSP, A | 3.4854 | 0.108 | 10 | 0.006 | ** |
| D, B | 1.4108 | 0.104 | 10 | 0.189 | ns |
| D, M | 1.6360 | 0.112 | 10 | 0.093 | ns |
| D, C | 1.7015 | 0.098 | 10 | 0.088 | ns |
| D, A | 3.9442 | 0.088 | 10 | 0.006 | ** |
| B, M | 1.0239 | 0.497 | 10 | 0.395 | ns |
| B, C | 1.3590 | 0.107 | 10 | 0.184 | ns |
| B, A | 2.1330 | 0.103 | 10 | 0.025 | * |
| M, C | 1.1934 | 0.217 | 10 | 0.251 | ns |
| M, A | 1.5932 | 0.109 | 10 | 0.100 | ns |
| C, A | 1.5273 | 0.189 | 10 | 0.134 | ns |
