# Supplementary material for: Biological contamination of macrobenthic communities in seminatural streams: implications for monitoring habitat quality in Isoëtes malinverniana stands
Source: Environ Monit Assess. 2026 Jul 31;198(8):896. doi: 10.1007/s10661-026-15738-8 (PMC13427863; doi:10.1007/s10661-026-15738-8)
Supplement: Supplementary file 2 — (DOCX 15.3 KB) [file 10661_2026_15738_MOESM2_ESM.docx]

**Supplementary Material 2.** Pairwise Montecarlo tests results considering only the presence of alien species. Ns: not significant; *: p<0.05, significant; **: p<0.005, very significant; ***: p<0.001, extremely significant. For the codes of the streams, see table 1

| Groups | t | p(perm) | perms | p(MC) | Statistical significance |
| --- | --- | --- | --- | --- | --- |
| SP594, D | 2.6458 | 0.333 | 2 | 0.056 | ns |
| SP594, M | 1.6957 | 0.203 | 10 | 0.107 | ns |
| SP594, C | 4.0629 | 0.106 | 5 | 0.008 | * |
| SP594, A | 2.9591 | 0.343 | 2 | 0.044 | * |
| D, M | 1,9433 | 0.094 | 7 | 0.056 | ns |
| D, C | 4.0629 | 0.123 | 5 | 0.011 | ** |
| D, A | 2.9591 | 0.337 | 2 | 0.055 | ns |
| M, C | 1.8157 | 0.089 | 7 | 0.078 | ns |
| M, A | 0.90643 | 0.79 | 10 | 0.458 | ns |
| C, A | 2.09 | 0.094 | 7 | 0.093 | ns |
